# Current State of Computed Tomography Derived Fractional Flow Reserve (FFR-CT) and Its Diagnostic Advantages

**DOI:** 10.1177/11795468261464355

**Published:** 2026-06-24

**Authors:** Ibrahim Mortada, Dalton Buckingham, Aaron W. Lee, Esosa Odigie-Okon, Mostafa Shalaby, Afaq Motiwala, Amer Abdulla, Michael C. Boyars, Thomas A. Blackwell, Hani Jneid

**Affiliations:** 1 Department of Cardiovascular Medicine, University of Texas Medical Branch, Galveston, TX, USA; 2 John Sealy School of Medicine, University of Texas Medical Branch, Galveston, TX, USA; 3 Department of Internal Medicine, University of Texas Medical Branch, Galveston, TX, USA

**Keywords:** fractional flow reserve, coronary CT angiography, coronary artery disease, FFR-CT

## Abstract

Coronary computed tomography angiography (CCTA) is widely used for noninvasive evaluation of coronary artery disease (CAD) and is highly sensitive for detecting anatomic coronary stenosis with a high negative predictive value. However, CCTA is limited in its ability to determine the physiological significance of lesions, resulting in reduced specificity and disagreement with invasive coronary angiography in a substantial proportion of cases. Fractional Flow Reserve derived from CCTA (FFR-CT) was developed to address this anatomic–physiologic discordance by providing noninvasive, lesion-specific functional assessment of ischemia. This narrative review summarizes the current state of FFR-CT technology, its diagnostic performance relative to CCTA alone and invasive FFR, and its evolving role in contemporary CAD evaluation. Across prospective trials and meta-analyses, FFR-CT consistently improves diagnostic accuracy for ischemia-producing lesions, driven primarily by gains in specificity, with favorable agreement to invasive FFR at clinically relevant thresholds. Advances in computational modeling and machine learning have substantially reduced processing times, improving feasibility and workflow integration. Clinical studies demonstrate that incorporation of FFR-CT following CCTA improves selection for invasive coronary angiography, reduces unnecessary diagnostic catheterization, and provides prognostic information beyond anatomic disease burden alone. Important limitations remain, including dependence on CCTA image quality, reduced reliability in heavily calcified or complex coronary anatomy, and uncertainty near ischemic thresholds, necessitating careful interpretation within the clinical context. When applied selectively after high-quality CCTA, FFR-CT offers a robust noninvasive surrogate for invasive coronary physiology and supports a more targeted, physiology-guided diagnostic pathway for patients with suspected CAD.

## Graphical abstract

**Graphical Abstract:**
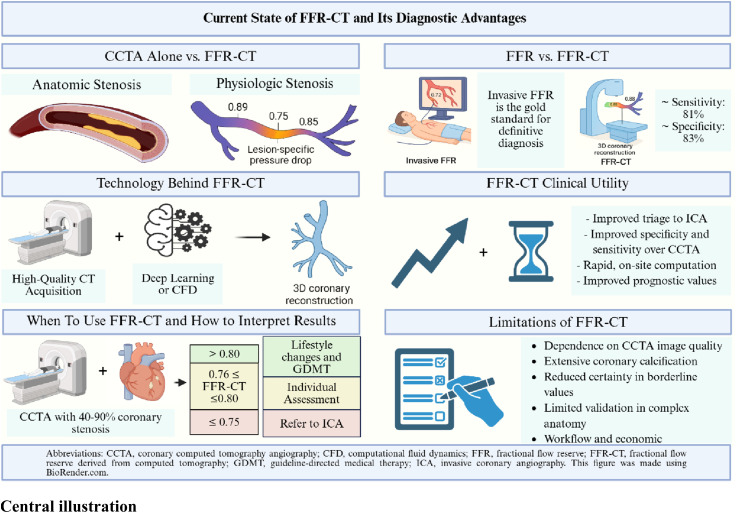

## Introduction

Coronary computed tomography angiography (CCTA) is a widely used, noninvasive tool for evaluating coronary artery disease (CAD) and is highly sensitive for identifying anatomic coronary stenosis and overall plaque burden.^1^ Like invasive coronary angiography, the technique’s principal limitation remains its imperfect ability to determine whether an anatomic stenosis produces physiologically significant ischemia. producing.^2,3^ This gap between anatomy and physiology reduces the specificity of CCTA and contributes to a substantial proportion of patients referred after abnormal CCTA with invasive coronary angiography (ICA) showing nonobstructive disease. Fractional Flow Reserve derived from CCTA (FFR-CT) was developed precisely to address this discordance by incorporating lesion-level physiological modeling into routine CCTA interpretation. By simulating hyperemic coronary flow through patient-specific CCTA-derived three-dimensional anatomic reconstructions, FFR-CT provides a functional correlate to purely anatomic assessment. For example, in DISCOVER-FLOW, the incorporation of FFR-CT significantly improved diagnostic accuracy relative to CCTA stenosis assessment alone, driven largely by gains in specificity.^2^ Subsequent investigations have shown that pairing FFR-CT with CCTA yields incremental diagnostic discrimination compared with anatomic imaging alone, including improvements in the area under the curve (AUC) for ischemia detection.^4^ The integration of anatomic and physiologic information within a single noninvasive test represents a meaningful advance in the diagnostic pathway for CAD and offers a more selective approach to determining which patients truly benefit from ICA. Accordingly, this review aims to synthesize the scientific foundations, evolving evidence base, and clinical implications of FFR-CT in the evaluation of coronary artery disease.

### Comparison With Invasive FFR

Invasive FFR remains the reference standard for assessing lesion-specific physiology, and any noninvasive alternative must therefore be judged by how closely it reflects this benchmark. Although FFR-CT shows a generally favorable correlation with invasive measurements, its clinical utility is better understood through its diagnostic performance rather than correlation coefficients alone. In a recent meta-analysis, FFR-CT demonstrated an overall diagnostic accuracy of 82.2%, with sensitivity and specificity of 80.9% and 83.1%, respectively.^5^ Building on these findings, contemporary analyses highlight that the most meaningful indicator of performance is how well FFR-CT classifies lesions around the invasive FFR threshold of ≤0.80 – a range where treatment decisions hinge on the likelihood of physiologically significant ischemia. Across multiple studies, FFR-CT has shown strong discriminatory ability in this critical zone, reinforcing its value as a functional complement to CCTA in guiding downstream management.^6^

The NXT trial (Analysis of Coronary Blood Flow Using CT Angiography: Next Steps) provided one of the clearest early validations of CT-derived fractional flow reserve using computational flow dynamics, showing that FFR-CT more accurately identified ischemia-producing lesions than CCTA alone, with AUCs of 0.90 and 0.81, respectively.^7^ In this prospective, multicenter study of 254 patients referred for invasive coronary angiography, FFR-CT demonstrated significantly higher diagnostic accuracy than anatomical CCTA by improving lesion-specific discrimination. This primarily occurred through marked gains in specificity – and correctly reclassified a substantial proportion of CCTA false positives, reinforcing its value in refining patient selection for invasive evaluation. The PACIFIC study (Prospective Head-to-Head Comparison of Coronary CT Angiography, SPECT, PET, and Hybrid Imaging for Diagnosis of Ischemic Heart Disease) further supported the diagnostic strength of FFR-CT when analyses were restricted to evaluable CCTA datasets. In these fully analyzable cases, FFR-CT demonstrated the highest per-vessel AUC (0.94) among all modalities. However, because about one quarter of the CCTA scans were non-evaluable, its diagnostic advantage lessened when all patients were considered in intention-to-diagnose analyses.^8^ Taken together, these studies highlight the importance of image quality and analytic feasibility in determining the reliability of FFR-CT in clinical practice.

Currently available technology also allows for rapid, machine learning-based approaches capable of generating results with markedly reduced processing time while maintaining strong agreement with invasive FFR.^9^ These technologies further support the conceptual feasibility of noninvasive physiologic assessment; however, longer-term outcome data and broader validation are still needed before assuming equivalence to invasive FFR. An additional nuance is that diagnostic certainty diminishes near the ischemic threshold. Borderline lesions with computed values in the 0.76–0.80 range may exhibit discordance with invasive FFR. In such cases, evaluation of the trans-lesional FFR-CT gradient can provide additional diagnostic clarity, as a gradient exceeding 0.12 has been associated with greater likelihood of physiologically significant disease and improved concordance with invasive indices.^10^ Despite these limitations, the body of evidence supports FFR-CT as a robust noninvasive surrogate for invasive FFR, offering a compelling physiologic complement to CCTA.^10^

### Technology Behind FFR-CT

Two distinct technological approaches are currently FDA-approved for FFR-CT: computational fluid dynamics (CFD)-based modeling and deep-learning based physiologic estimation. The CFD-based platform (HeartFlow) constructs a patient-specific three-dimensional coronary model and applies physiologic fluid-dynamic simulations grounded in three key assumptions: that resting coronary blood flow scales with myocardial mass, that microvascular resistance varies inversely with luminal size, and that the vasodilatory response to adenosine is predictable. These principles allow the model to estimate pressure loss and flow limitation under simulated hyperemia.^3^ In contrast, DEEPVESSEL FFR (Keya Medical) employs an end-to-end deep learning framework to analyze CCTA datasets, construct a coronary vessel model, and predict FFR values through data-driven learning of the relationship between coronary anatomy and FFR values, rather than explicit physics-based fluid simulation.^11^ The two platforms differ in several practically important ways. CFD-based approaches derive from a well-established physics-based framework with the largest body of prospective multicenter validation and regulatory clearance data. Deep learning–based methods substantially reduce computation time – from the 1–5 hours typically required for off-site CFD analysis to results available in minutes on-site – which alleviates a major workflow limitation of the CFD approach.^9,12^ However, deep learning platforms have a comparatively smaller prospective validation evidence base, and direct head-to-head comparisons between currently approved platforms remain limited. Based on currently available data, no strong conclusions can be drawn about diagnostic equivalence between CFD and deep learning-based FFR-CT; the choice between platforms should therefore be guided by local availability, CCTA image quality, and the strength of validation data applicable to the clinical scenario at hand.^9,13^ Together, these two FDA-approved approaches reflect the evolution of FFR-CT technology toward faster, more accessible computation while preserving the goal of reproducing lesion-specific physiology noninvasively.

The computation of FFR-CT begins with high-quality CCTA acquisition, from which patient-specific three-dimensional whole heart and coronary lumen geometries are derived. Automated or semi-automated segmentation tools extract the coronary centerlines and vessel boundaries, constructing a three-dimensional anatomic model.^14^ Using this model as a framework, physiologic assumptions are applied to simulate hyperemic flow conditions and estimate pressure differentials across coronary lesions. These simulations rely on computational fluid dynamics (CFD) or hybrid reduced-order models that incorporate equations governing fluid behavior, vessel compliance, and microvascular resistance.^13,14^ Although CFD-based analyses are computationally demanding, ongoing refinements benefitting from larger datasets – such as deep learning-assisted segmentation and reduced-order physiologic modeling – have markedly improved efficiency.^12^ Emerging AI-based platforms increasingly combine CT-derived physiological assessment with automated plaque characterization, enabling integrated interpretation of stenosis severity, plaque features, and vessel-specific ischemic risk. These multimodal approaches hold promise for the more personalized risk stratification, though broader prospective validation is needed.^13^

Consequently, the accuracy of FFR-CT is intimately tied to the quality of CCTA acquisition. Motion artifacts, elevated heart rate, high body mass index, and extensive calcification all challenge precise lumen boundary extraction and can alter the downstream physiologic model.^13^

In multicenter studies such as PACIFIC, non-evaluable scans represented a substantial fraction of the study population and meaningfully influenced intention-to-diagnose performance.^8^ Platforms that perform on-site FFR-CT calculations, often within minutes of image acquisition, have the potential to streamline workflow and minimize logistical delays associated with off-site analysis.^9^ These developments improve feasibility but do not eliminate the need for rigorous acquisition protocols and thoughtful integration with clinical and anatomic findings.

Additionally, workflow innovations increasingly emphasize computational speed and local availability. The on-site deep learning model evaluated by Giannopoulos and colleagues reduced mean analysis time to under eight minutes while maintaining strong agreement with invasive indices in a small cohort.^9^ This suggests that logistical barriers (off-site transfer and long turnaround) may be less limiting over time, but it does not change the central requirements for high-quality acquisition, careful interpretation, and appropriate patient selection.^9,15^

### FFR-CT Clinical Utility

The main clinical value of FFR-CT is its ability to guide further management following CCTA by providing lesion-specific physiological information on ischemia. Contemporary evidence demonstrates that incorporation of FFR-CT into CCTA-based evaluation improves selection for ICA, particularly in patients with anatomically intermediate disease, leading to fewer ICAs overall and a lower proportion of catheterizations demonstrating non-obstructive coronary arteries without an increase in adverse clinical events.^16-18^

Clinical utility has also been demonstrated with respect to patient-centered outcomes. Abnormal FFR-CT values are associated with higher rates of recurrent angina and increased symptom-driven healthcare utilization, whereas preserved FFR-CT is associated with fewer recurrent symptoms, suggesting that computed physiology captures clinically meaningful ischemia beyond anatomic stenosis severity alone.^19,20^ In addition, lower FFR-CT values have been independently associated with worse longer-term cardiovascular outcomes after adjustment for anatomic disease burden, supporting a role for FFR-CT in prognostic risk stratification in addition to procedural triage.^21^

Machine-learning-based and on-site FFR-CT approaches have demonstrated substantially reduced processing times while maintaining agreement with invasive physiologic indices, which alleviates previous workflow limitations of off-site analysis.^9,22^

Available evidence also provides insight into how FFR-CT diagnostic performance varies by stenosis severity and lesion morphology, though the data are not uniform across studies. Diagnostic certainty is most reduced in the borderline stenosis range (approximately 0.76–0.80), where agreement with invasive FFR is consistently at its lowest across multiple cohorts.^5,6^ Certain analyses have identified a trend toward greater numerical discordance between CT-FFR and invasive FFR measurements as coronary calcium burden increases, and have found that heavily calcified lesions carry a higher likelihood of spuriously ischemic FFR-CT readings compared with non-calcified counterparts.^5^ In contrast, prospective multicenter evidence from the CT-FFR CHINA (Computed Tomography Derived Fractional Flow Reserve for Coronary Hemodynamic Ischemia Noninvasive Assessment) trial found that overall diagnostic performance, assessed at both the patient and vessel level, remained statistically comparable across the full range of coronary artery calcium scores, implying that coronary calcification may carry a smaller influence on physiologic FFR-CT analysis than on the anatomic stenosis characterization provided by CCTA alone.^23,24^ These observations collectively reinforce that FFR-CT is most reliable when applied to intermediate-stenosis populations where physiologic clarification is clinically meaningful.

### When to Use FFR-CT and How to Interpret Results

In contemporary practice, FFR-CT is most appropriately applied following CCTA when anatomic findings alone do not adequately define the physiologic significance of coronary lesions. Its greatest value lies in patients with intermediate or ambiguous stenoses, where the relationship between luminal narrowing and ischemia is most variable and where physiologic assessment can meaningfully alter management decisions.^25^ Consistent with this evidence, the 2021 ACC/AHA Chest Pain Guideline designates FFR-CT as a Class IIa recommendation for evaluating vessel-specific ischemia in patients with intermediate coronary stenoses of 40–90% identified on CCTA.^26^

Interpretation of FFR-CT generally parallels invasive FFR, with values ≤0.80 considered suggestive of ischemia-producing physiology.^27^ A single distal FFR-CT value may not fully reflect physiologic risk. Evaluating how pressure drops along the vessel, particularly across a specific lesion, can add clinically useful information and help identify functionally significant disease, even when distal FFR-CT values are borderline.^28^ Borderline FFR-CT values should therefore be interpreted in the context of symptom burden, plaque morphology, lesion location, and overall cardiovascular risk. In such cases, FFR-CT should be viewed as complementary rather than definitive, and additional functional testing or invasive evaluation may remain appropriate.^10,15^ Technical limitations, including motion artifact, extensive coronary calcification, or complex post-revascularization anatomy, further reinforce the importance of careful patient selection and high-quality CCTA acquisition. Across systematic analyses, FFR-CT has demonstrated overall diagnostic accuracy in the range of approximately 80% compared with invasive FFR, although performance varies widely across the spectrum of FFR-CT values and clinical settings, with lower specificity and predictive certainty observed in intermediate stenoses.^5,24^ Real-world audits have shown positive predictive value near ∼50%, underscoring the influence of image quality, referral patterns, and pretest probability on test performance.^29,30^ Despite substantial advances in computational speed and clinical integration, the overall diagnostic accuracy of FFR-CT compared with invasive FFR has remained relatively stable since early validation studies in 2014 with the NXT Trial, with contemporary meta-analyses reporting an accuracy of approximately 80%.^5,31^

Beyond its established role in evaluating intermediate coronary stenoses, emerging evidence supports the use of FFR-CT in preoperative planning for coronary artery bypass grafting (CABG) and in selected vascular surgery populations, where physiologic clarification of coronary disease can improve preoperative risk assessment. In a retrospective multicenter study, preoperative FFR-CT values ≤0.80 were associated with a significantly lower risk of subsequent graft failure following CABG, suggesting that physiologic assessment of target vessels may help refine graft selection and reduce graft failure. Surgical decision-making based on combined CCTA and FFR-CT has also been shown to be feasible, with a panel of cardiac surgeons demonstrating excellent agreement (intraclass correlation coefficient 0.77) on the appropriate number of anastomoses in 84% of cases.^32^ These findings highlight the expanding utility of FFR-CT beyond diagnostic triage, underscoring its potential value in optimizing revascularization strategy and improving long-term graft outcomes.^33-38^

### What do Guidelines Recommend Regarding FFR-CT

Contemporary guidelines recognize FFR-CT as a physiologic adjunct to CCTA within structured diagnostic pathways for stable CAD. The 2021 ACC/AHA Chest Pain Guideline identifies CCTA as an effective front-line test in patients with intermediate-to-high pretest probability and no known CAD, serving simultaneously as a diagnostic tool, a means of risk stratification, and a guide for selecting appropriate downstream management. When CCTA identifies coronary stenoses in the 40–90% range, the guideline assigns FFR-CT a Class IIa recommendation, highlighting its usefulness in determining vessel-specific ischemia and informing decisions regarding invasive angiography or revascularization. This approach reflects the guideline’s broader emphasis on integrating physiologic assessment with anatomic imaging to refine patient selection and enhance the efficiency of chest pain evaluation pathways.^26^ The 2024 European Society of Cardiology Guidelines for Chronic Coronary Syndromes emphasize integration of anatomic and functional information to improve diagnostic specificity and optimize referral for ICA, noting that physiologic assessment following CCTA can reduce unnecessary invasive testing when image quality is adequate and stenosis severity is uncertain.^17^ Health technology assessments and expert consensus documents similarly support the conditional use of FFR-CT after CCTA in patients with stable symptoms and intermediate lesions when physiologic clarification is expected to influence management. NICE guidance endorses FFR-CT as an option within post-CCTA diagnostic pathways to improve patient selection for invasive evaluation, particularly by reducing unnecessary angiography.^39^ Expert consensus statements further emphasize that FFR-CT is most valuable when resolving anatomic-physiologic discordance rather than as a routine adjunct to all CCTA examinations.^40^

**Figure 1.**
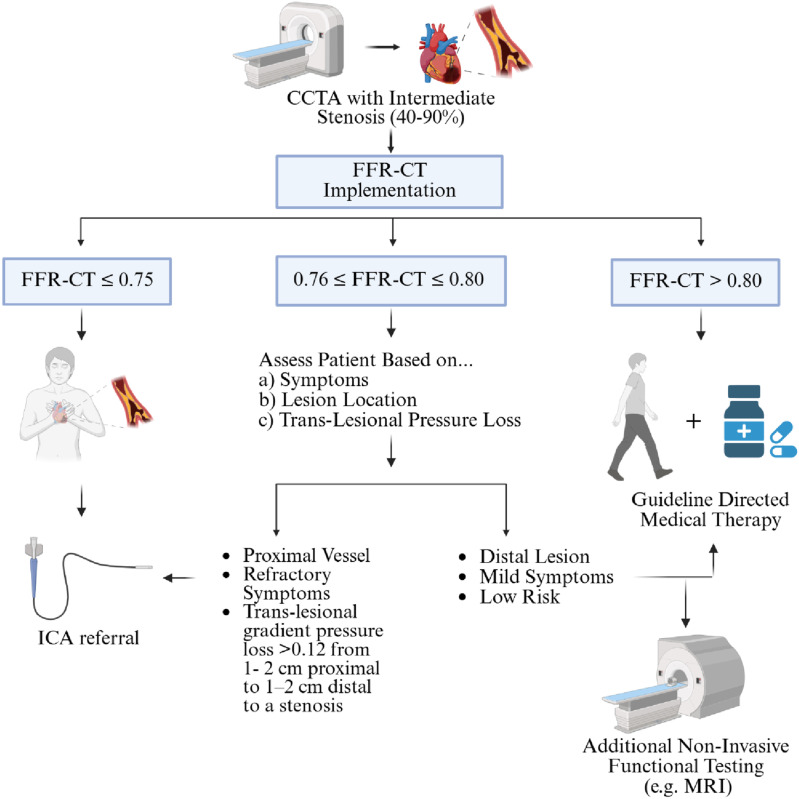
Clinical decision pathway for the use and interpretation of FFR-CT following coronary CT angiography. Abbreviations: CCTA, Coronary computed tomography angiography; FFR-CT, Fractional flow reserve derived from CCTA; ICA, Invasive coronary angiography; MRI, Magnetic resonance imaging

Across guideline and consensus documents, a consistent theme emerges: FFR-CT is best positioned as a selective, physiology-guided extension of CCTA that complements clinical judgement within a tiered diagnostic strategy. This positioning aligns with broader effort to improve precision in noninvasive coronary evaluation by targeting invasive testing to patients most likely to benefit while minimizing unnecessary procedures (Figure 1). Major guidelines recommendations on FFR-CT usage are summarized in Table 1.

**Table 1.** Society Guideline Recommendations on FFR-CT Usage

Guideline/Organization	Population (age/risk)	Next step based on FFR-CT result
CAD-RADS™ 2.0 (2022)^41^	Coronary stenosis ranging from 50-90% (particularly CAD-RADS 3 and 4a).	Non-ischemic; Normal (>0.80): Defer ICA; optimize medical therapy.
		Borderline (0.76–0.80): Consider ICA based on symptoms, lesion location, and trans-lesional pressure loss.
		Ischemic; Abnormal (≤0.75): Consider ICA ± revascularization for individuals likely to benefit.
ACC/AHA Chest Pain Guideline (2021)^26^	Patients with obstructive CAD (≥50% stenosis) or inconclusive stenosis on CCTA; favored in patients <65.	Ischemic (≤0.80): ICA/revascularization consideration.
		Non-ischemic (>0.80): GDMT; defer ICA.
NICE HTG429 — HeartFlow FFRCT (2021)^39^	Stable, recent-onset chest pain undergoing CCTA; no age cutoffs; requires ≥64-slice CT.	Ischemic (≤0.80): ICA referral.
		Non-ischemic (>0.80): Avoid ICA; manage non-invasively.
SCCT Expert Consensus (2021)^40^	Intermediate (30–70%) stenosis on CCTA; CCTA less appropriate in very low-risk <40y.	Ischemic (≤0.80): ICA referral.
		Non-ischemic (>0.80): Avoid ICA; medical therapy.
ESC Chronic Coronary Syndromes (2024)^17^	Patients with suspected CCS undergoing stepwise evaluation with CCTA as first-line anatomic testing.	When anatomy inconclusive: CT-based functional imaging may clarify stenosis significance.
	CCTA is not recommended in patients with severe renal failure (eGFR <30ml/min/1.73 *m*2*m2*), decompensated heart failure, extensive coronary calcification, allergy to iodinated contrast, severe obesity, or any other conditions that can negatively impact quality of good imaging.	If ischemia demonstrated (≤0.80): Consider ICA and revascularization strategy.

**Abbreviations:** CCS, Chronic coronary syndromes; GDMT, Guideline-directed medical therapy; ICA, Invasive coronary angiography.

### Limitations of FFR-CT

The robustness of FFR-CT faces multiple constraints that limit its widespread application despite proven diagnostic value. Image quality dependency represents a fundamental limitation, as diagnostic performance is heavily compromised by motion artifacts (the leading cause of rejection, accounting for 64-78% of failed cases), extensive coronary calcification, metallic artifacts from stents or cardiac devices, misalignment, noise, and inadequate contrast opacification, resulting in 3-33% of CT examinations being unsuitable for analysis.^1,15,42-44^ However, emerging photon-counting CT technology helps mitigate one of these major limitations by substantially reducing the blooming and luminance distortion caused by extensive coronary calcification, thereby improving the reliability of lumen assessment in heavily calcified vessels.^45,46^ Emerging data are beginning to address whether photon-counting CT improves FFR-CT reliability in practice. Portolan and colleagues evaluated both standard and ultra-high-resolution photon-counting CCTA (UHR-PCCTA)-derived FFR values against invasive FFR measurements. Early evidence supports that the superior spatial resolution and diminished blooming artifact characteristic of photon-counting detectors may enable more accurate coronary lumen characterization and FFR-CT estimation in vessels with substantial calcification – the subset of patients in whom the accuracy of conventional CCTA-based FFR-CT analysis is most limited.^45-47^ These findings are preliminary and prospective multicenter validation is needed before UHR-PCCTA-derived FFR can be recommended broadly, but the technology represents a meaningful avenue for expanding the proportion of patients in whom reliable physiologic assessment can be performed noninvasively. Technical specifications further restrict applicability, including requirements for complete cardiac coverage, slice thickness below 1 mm, appropriate spacing and pixel dimensions, and continuous datasets without missing sections.^15^ The technology remains unvalidated in several important clinical scenarios, including post-PCI patients, those with myocardial bridges, complex bifurcations, coronary stents, bypass grafts, and acute coronary syndromes, necessitating further investigation to define appropriate clinical indications.^1,15,42^ Computational fluid dynamics-based platforms require prolonged processing times of 1-5 hours, limiting utility in acute settings, though machine learning approaches promise faster on-site computation but lack validation and regulatory clearance.^42,43^ Economic considerations also influence the clinical adoption of FFR-CT, although specific costs vary widely across health systems and payer structures and therefore cannot be uniformly quantified. Real-world evidence from the National Health Service in England suggests that incorporating FFR-CT into CCTA-based pathways can reduce downstream diagnostic testing and invasive angiography, supporting the potential for meaningful system-level cost savings without compromising clinical outcomes.^48^

## Conclusion

FFR-CT has emerged as a valuable physiologic complement to anatomic imaging, addressing a key limitation of CCTA by clarifying the ischemic significance of coronary stenoses. Available evidence demonstrates that FFR-CT improves diagnostic specificity, refines selection for ICA, and provides prognostic information beyond luminal stenosis severity alone, with the greatest clinical impact observed in patients with intermediate or ambiguous anatomic disease. Technological advances, including machine learning–based modeling and on-site computation, have improved analytic speed and feasibility, supporting broader clinical integration. Consistent with contemporary guidelines and consensus recommendations, FFR-CT is best positioned as a selective, physiology-guided extension of CCTA rather than a routine adjunct, and when applied judiciously after high-quality CCTA imaging, it represents the most robust noninvasive surrogate for invasive coronary physiology currently available, enabling a more precise and patient-centered diagnostic pathway for stable coronary artery disease.
